# An Open-Label, Randomized Field Trial Demonstrates Safety and Immunogenicity of Inactivated gE-Deleted Marker Vaccine Against Infectious Bovine Rhinotracheitis in Cattle

**DOI:** 10.3390/vaccines13060579

**Published:** 2025-05-29

**Authors:** Bhaskar Ganguly, Sarvesh Tayshete, Deepa Padinjare Melepat, Sudhakar Awandkar, Srinivas Karnati, Priyabrata Pattnaik, Anand Kumar Kanakasapapathy

**Affiliations:** 1 Indian Immunologicals Limited, Rakshapuram 500032, Telangana, India; 2 Department of Veterinary Epidemiology and Preventive Medicine, College of Veterinary and Animal Science, Pookode 673576, Kerala, India; 3 Department of Veterinary Medicine, College of Veterinary and Animal Science, Udgir 413517, Maharashtra, India

**Keywords:** bovine herpesvirus-1, infectious bovine rhinotracheitis, vaccine, safety, immunogenicity

## Abstract

**Background**: Infectious Bovine Rhinotracheitis (IBR), Infectious Pustular Balanoposthitis (IPB), Infectious Pustular Vulvovaginitis (IPV), late-term abortions, and neurological and systemic disease are common manifestations of Bovine Herpesvirus-1 (BoHV-1) infections. IBR is enzootic to India and several other countries across the world. Globally, both live attenuated and inactivated vaccines are available commercially for the control of the disease. This communication reports the results of an open-label, randomized field trial of an inactivated IBR marker vaccine in cattle. **Methods**: An indigenously developed, inactivated, glycoprotein-E (gE)-deleted marker vaccine was subjected to a field trial involving 90 healthy cattle of more than three months of age, evaluating its safety and immunogenicity. **Results**: Vaccination was safe without any adverse and serious adverse events, except a self-limiting and self-subsiding induration at the site of injection in a few cases. The vaccine caused elevation of body temperature but within normal physiological range; no derangements in feed intake or milk yield were recorded. A total of 90% of the subjects developed protective titers of SNT_50_ ≥ 8 after receiving both doses of initial vaccination and maintained protective titers until 180 days thereafter. **Conclusions**: Altogether, our findings uphold that the indigenously developed, inactivated gE-deleted marker vaccine against IBR is safe and results in protective levels of immunity for at least six months in cattle of more than three months of age.

## 1. Introduction

The appearance of a respiratory disease in the USA characterized by sudden onset, high fever, and severe agalactia between the years 1950 to 1954 [1] brought specific recognition to Infectious Bovine Rhinotracheitis (IBR). However, similar diseases had been reported in European cattle along with demonstration of an underlying viral etiology many years before [2,3]. The disease has gained worldwide importance over the years, and the causative virus has been named Bovine Herpesvirus-1 (BoHV-1). Subsequently, the viral agent has been associated with two other major syndromes, viz. Infectious Pustular Balanoposthitis (IPB) and Infectious Pustular Vulvovaginitis (IPV), and also causing other clinical syndromes such as abortion, infertility, conjunctivitis, encephalitis, enteritis, and fatal systemic infection in neonatal calves [4,5].

The presence of the virus in bovine populations has been associated very strongly with bovine respiratory disease complex, abortions, and infertility [6,7,8], all of which cause severe economic losses, compelling countries to take up nationwide eradication programs [9]. The disease has been estimated to cost approximately USD 3 billion in losses to the cattle industry every year [10]. Countries pursuing systematic disease eradication programs have adopted repeated administration of inactivated vaccines to achieve significant control over the disease; the use of live vaccines allows disease control but is neither safe nor amenable to disease eradication programs [11,12].

IBR is enzootic to India, and the need for a nationwide vaccination program with an inactivated marker vaccine has long been recognized [13,14]. Currently, no indigenous vaccine is available in India against the disease, and only one imported vaccine is authorized for sale in Indian markets, which has proven limiting in the pursuit of a national eradication program due to cost and availability. This communication reports the results of a field trial on the assessment of the safety and immunogenicity of India’s first indigenous vaccine against IBR developed by *M*/*s* Indian Immunologicals Limited (IIL) [15,16]. The primary objectives of this study were to evaluate the safety of inactivated IBR recombinant vaccine and to observe the frequency of adverse and serious adverse events in vaccinated animals in comparison with unvaccinated controls. The secondary objectives of the study included the determination of immune response to the vaccine and the effect of the vaccine on the milk yield of lactating animals.

## 2. Material and Methods

### 2.1. Trial Design

An open-label, randomized field trial was undertaken to evaluate the safety and immunogenicity of an inactivated gE-deleted IBR vaccine in five groups of healthy cattle, viz. male calves, female calves, non-lactating cows, lactating cows, and breeding bulls. The trial was replicated at two geographically varying trial sites, viz. Site A, i.e., College of Veterinary and Animal Sciences (CoVAS), Udgir (18.3943° N, 77.1126° E, 646 msl), and Site B, i.e., College of Veterinary and Animal Sciences (CoVAS), Pookode (11.5412° N, 76.0218° E, 1083 msl), from October 2022 to October 2023, including the period of screening. The trial protocol was approved by the Drugs Controller General of India (DCGI) and the Institutional Animal Ethics Committees (IAEC) of the respective trial sites. The trial was conducted in compliance with VICH GL9 GCP guidelines [17]. At each trial site, the subjects were assigned to the treatment or control arm in a sequence as per the randomization list prepared by an independent statistician at a Contract Research Organization, and the vaccine or control was administered by the designated study personnel as per the randomization codes.

### 2.2. Study Population

The study subjects included healthy cattle of either sex, aged 4 months to 15 years at screening, and seronegative, i.e., median serum neutralization titers (SNT_50_) < 2, for anti-BoHV-1 antibodies. The main exclusion criteria were planned participation in any other trial within four weeks preceding or during the present trial period; previous vaccination or evidence of infection with BoHV-1; pregnancy; history of allergy or reaction to vaccine components; intercurrent illness or chronic disease; and any other conditions that may pose a health risk or interfere with the evaluation of the vaccine response.

### 2.3. Sample Size Calculation

Based on the assumptions of baseline prevalence ≥40% in the population, incidence in the vaccinated group ≤5%, vaccine: control enrolment ratio = 2:1, confidence = 95%, and power = 0.8, the number of animals was calculated at 37 in the vaccine group and 19 in the control group, which was further increased to 45 animals in the vaccine group and 22 animals in the control group upon accommodating 15% drop-outs and lost to follow-up. The animals were further divided into five physiological groups of male calves, female calves, dry cows, lactating cows, and breeding bulls at each trial site, as shown in Table 1, and these numbers were replicated at each of the two trial sites.

### 2.4. Test Substances and Vaccination Schedule

**Figure 1:**
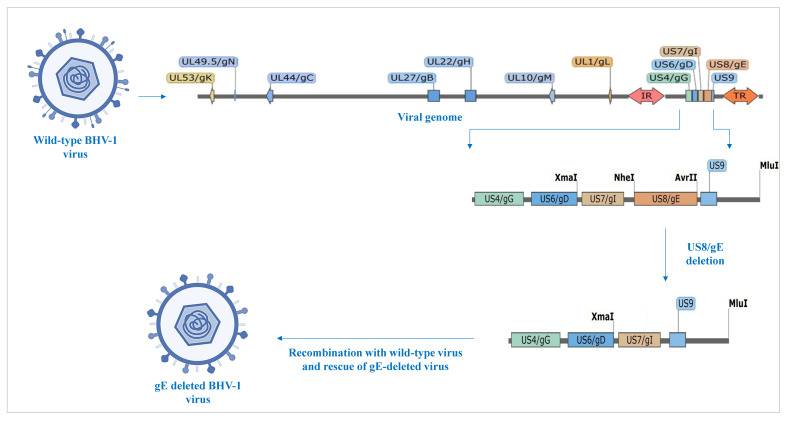
Glycoprotein-E-deleted viral construct used in vaccine.

The inactivated Infectious Bovine Rhinotracheitis vaccine (Figure 1), indigenously developed at IIL by deleting the glycoprotein-E gene from wild-type virus isolated in India [15,16], was used in the study. Each dose of the test vaccine contained >10^8^ TCID_50_ of BoHV-1 (titer before inactivation), 0.5 mL of 2% *w*/*v* aluminum hydroxide, 0.002 mL of 10% *w*/*v* thiomersal, and 2% *w*/*v* phosphate-buffered diluent *q.s.* to 2.0 mL. Batch number 01IBREXP00222 of the vaccine was used in the trial. The vaccine doses were stored at 2–8 °C until administration.

The subjects in the treatment arms received primary vaccination with two doses of the vaccine at an interval of 30 days and revaccination with another dose of the vaccine after 180 days of the first dose. The subjects in the control arm received 2.0 mL of 2% (*w*/*v*) phosphate-buffered diluent on the corresponding occasions. The vaccine or control was administered intramuscularly in the mid-neck region through an area of clean, dry skin following standard aseptic procedures.

### 2.5. Safety

All the animals were observed intensively for local and systemic reactions after each vaccination on days 0, 30, and 180. Rectal temperature was recorded four times at 0, 3, 6, and 12 h on the first day and once daily for another fourteen days after each of the three vaccinations on days 0, 30, and 180. Feed intake was calculated by deducting leftover feed from the offered feed daily and recorded for seven days after each of the three vaccinations on days 0, 30, and 180. Similarly, the milk yield of lactating animals was recorded five days before and nine days after each of the three vaccinations on days 0, 30, and 180.

### 2.6. Immunogenicity

Blood samples were collected by jugular venepuncture on days 0, 30, 60, 180, and 210 for estimation of virus neutralizing antibody titers by Serum Neutralization Test (SNT). SNT was performed as per the WOAH [18] using a constant virus diluting serum protocol against a wild-type virus, and titers were expressed as the reciprocal of the highest serum dilution that prevented the induction of cytopathic effects up to after 5 days of incubation.

Group-wise and cumulative geometric mean titers, seroconversion rates, and seroprotection rates of the vaccine were calculated and compared with the respective controls. Seroconversion was defined as a four-fold rise in SNT_50_ on day 60. SNT_50_ ≥ 8 on day 60 was used as the cut-off for protection [19]. The pass criterion for the immunogenicity assessment was set as seroprotection in ≥80% of the vaccinated subjects on day 60 [20].

### 2.7. Statistical Analyses

Statistical significance was determined by one-way ANOVA, using Excel for Microsoft 365 (Microsoft Corp, Redmond, WA, USA) with XL Toolbox add-in version 7.3.4 [21]. Unless stated otherwise, significance was inferred at *p* ≤ 0.05.

## 3. Results

In total, 134 animals, i.e., 67 animals at each of the two trial sites, were recruited in the study. A total of 103 animals were screened at Site A of which 11 animals, having titers ≥ 2, were excluded as seropositive. A total of 67 of the remaining 92 seronegative animals were recruited in the study. At Site B, 77 animals were screened, of which 10 animals, having titers ≥ 2, were excluded as seropositive. All the remaining 67 seronegative animals were recruited in the study. There were no drop-outs, and all 134 recruited animals completed the study.

The recruited animals received 02 mL of the vaccine or control on days 0, 30, and 180 of the trial. In general, the vaccine was well tolerated by all the animals. Site A reported a self-limiting, mild induration at the site of vaccine injection in four (04) of 135 (45 animals × 3 doses) instances, which subsided uneventfully within 24 h without any medication. Site A did not report any other solicited or unsolicited adverse events or serious adverse events. Site B did not report any solicited or unsolicited adverse events or serious adverse events.

**Figure 2:**
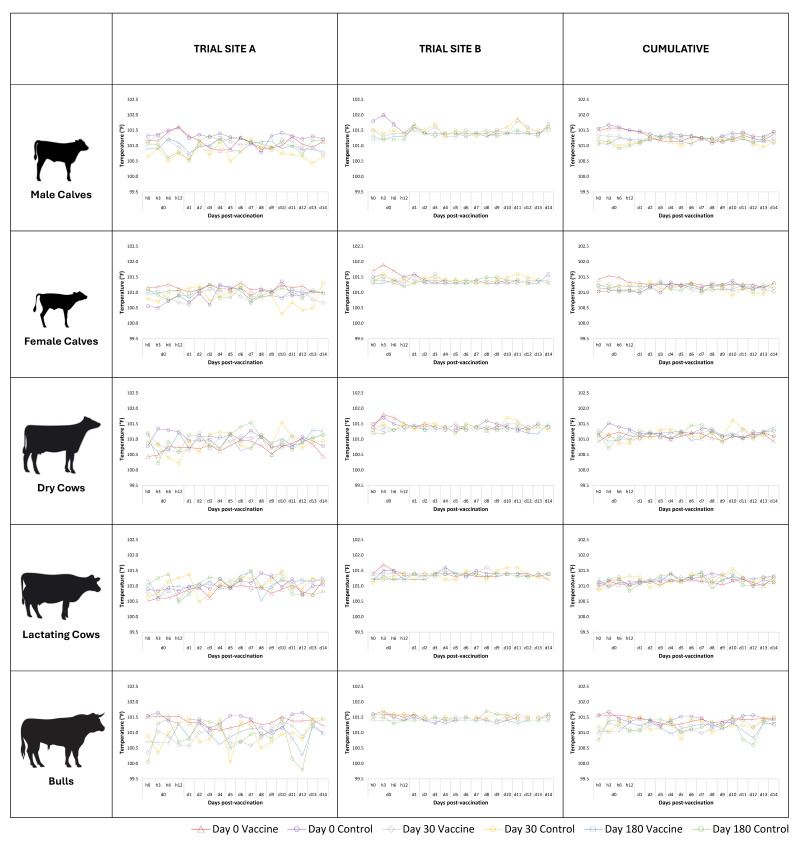
Site-wise and group-wise mean body temperature of subjects. Rectal temperature (°F) was recorded four times at 0, 3, 6, and 12 h on the day of vaccination and once daily for another fourteen days after each of the three vaccinations on days 0, 30, and 180. Despite fluctuations, the body temperature of all subjects remained well below 102.5 °F at all the observation time-points.

Mean body temperatures have been shown in Figure 2 for comparison. Most groups of animals showed a transient increase in body temperature during the first 24 h following vaccination; this increase was higher after the first dose of the vaccine as compared to the two subsequent doses of the vaccine. At Site A, all groups, viz. male calves (*p* << 0.01), female calves (*p* << 0.01), dry cows (*p* = 0.04), lactating cows (*p* = 0.04), and bulls (*p* << 0.01), showed a significant increase in body temperature post-vaccination. At Site B, male calves (*p* = 0.02), dry cows (*p* = 0.01), and bulls (*p* << 0.01) showed a significant increase in body temperature post-vaccination. Cumulatively, significant rises in body temperature post-vaccination were observed in male calves (*p* << 0.01), female calves (*p* << 0.01), and bulls (*p* << 0.01); within calves, the rise was greater in males than in females. Between sites, the corresponding group at Site A showed a greater increase in body temperature post-vaccination than the group at Site B (*p* << 0.01). However, at both Sites A and B, the body temperature of all subjects remained well below 102.5 °F at all the observation time-points.

**Figure 3:**
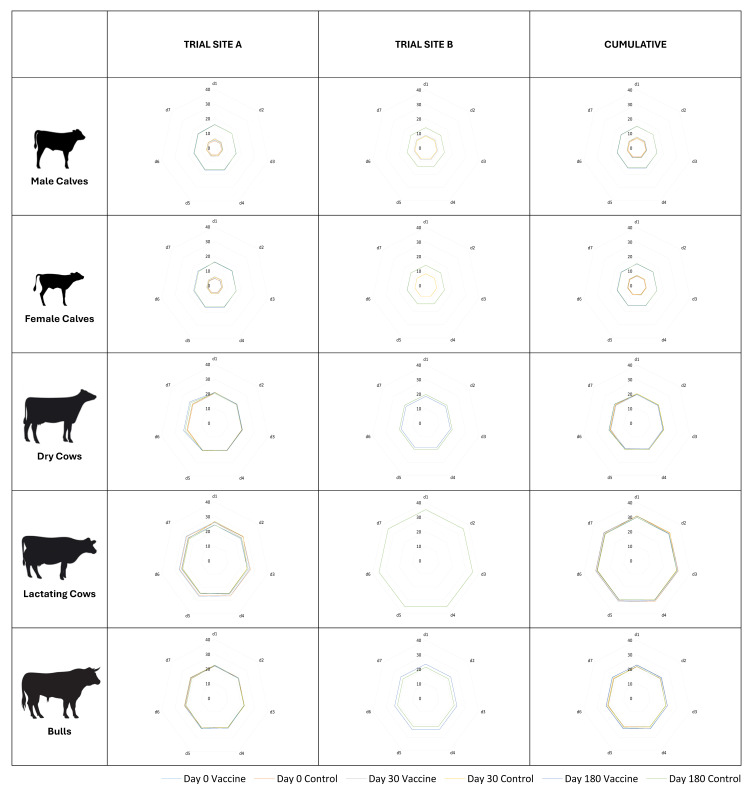
Site-wise and group-wise mean feed intake of subjects. Feed intake (kg/animal/day) was recorded for seven days after each of the three vaccinations on days 0, 30, and 180. A transient, non-significant decrease in feed intake was recorded for up to three days following vaccination at Site A only; no such changes were apparent at Site B.

Group-wise mean daily feed intakes are shown in Figure 3. Neither Site A nor Site B recorded any significant differences (*p* >> 0.05) in the mean intakes of the vaccinated animals in comparison to the unvaccinated controls in any of the groups following any of the three doses. A transient, non-significant decrease in feed intake, averaging less than 0.5 kg, was recorded between days 1 and 3 following vaccination in all the groups, viz. male calves, female calves, dry cows, lactating cows, and bulls of Site A only. The intake of feed was restored to normal quickly and completely by day 5 of vaccination. Such changes in feed intake were not apparent in the groups at Site B.

**Figure 4:**
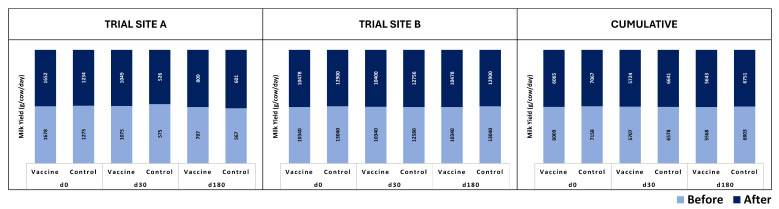
Site-wise daily milk production in lactating cows. The daily milk yield (g/cow/day) of lactating animals was recorded for five days before and nine days after each of the three vaccinations on days 0, 30, and 180, and changes in yield before and after vaccination were compared. No significant changes in milk yield were recorded at either of the sites or cumulatively.

Milk yield in the lactating cows for 5 days before and 9 days after vaccination was compared to evaluate the impact of the vaccine on milk production (Figure 4). At Site A, the mean milk yield per cow per day over five days before the first dose of primary vaccination was 1678 g, and over nine days following vaccination was 1652 g. With the second dose of primary vaccination, the mean milk yield per cow per day over five days before vaccination was 1075 g, and over nine days following vaccination was 1049 g, respectively. Three (03) animals in the vaccine arm became dry by this time-point. With the third dose, i.e., revaccination, the mean milk yield per cow per day over five days before vaccination was 797 g, and over nine days following vaccination was 809 g, respectively. Four (04) animals in the vaccine arm became dry by this time-point. At Site B, the average daily milk yield per cow was recorded to be greater in the treatment arm after (10,478 g) than before (10,340 g) the first vaccination. Similarly, the average daily milk yield per cow was again recorded to be greater in the treatment arm after (10,478 g) than before (10,340 g) the third vaccination. These differences in the milk yield before and after vaccination were not statistically significant at Site A (*p* = 0.19), Site B (*p* = 0.54), or cumulatively (*p* = 0.70).

None of the lactating or dry cows were pregnant at the time of receiving the first dose of the vaccine. However, by the time of receiving the second dose of the vaccine, two dry cows had become pregnant at Site A, and one lactating cow had become pregnant at Site B. Similarly, at the time of receiving the third dose of the vaccine, another two dry cows had become pregnant at Site A and another four lactating cows, and one dry cow had become pregnant at Site B. Hence, in all, there were thirteen instances of the vaccine being administered during early to late pregnancy. No gestational accidents or other adverse events were recorded in any of these instances. Although the sample size was small to draw meaningful inferences, any differences in conception rates between the cows of the vaccine and control arms were not apparent.

**Figure 5:**
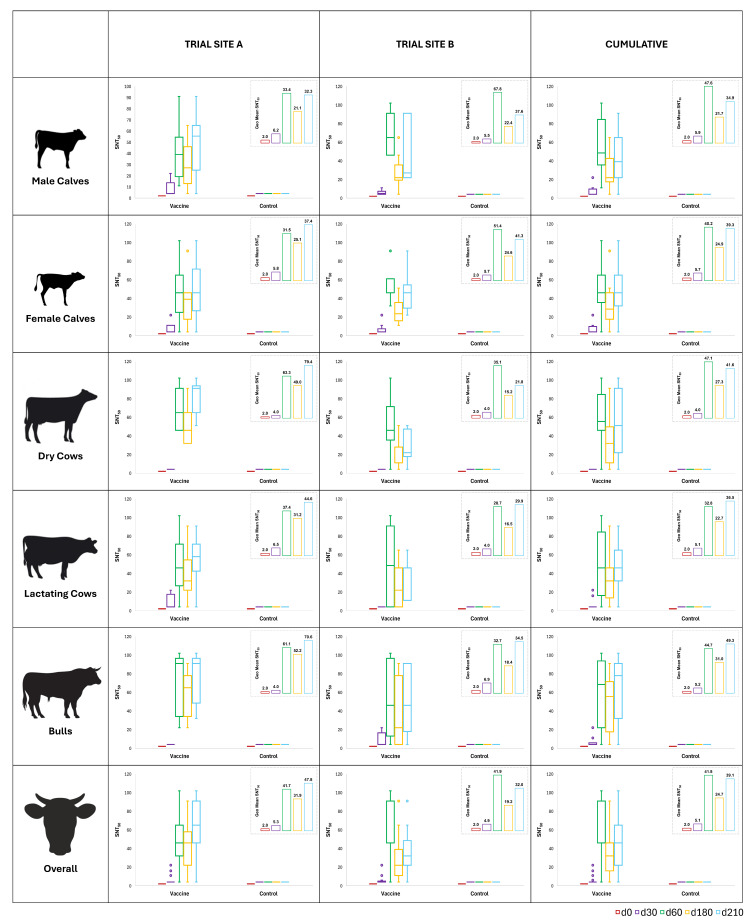
Site-wise and group-wise distribution of SNT_50_. Median serum neutralization titers were measured on days 0, 30, 60, 180, and 210. The distribution of the titers is shown as a box and whisker plot. Inset shows the geometric mean SNT_50_ of the vaccine arm only. Peak titers were observed at day 60, corresponding to 4 weeks after both doses of the primary vaccination. The titers decreased substantially by day 180, still persisting above protective levels, and spiked markedly by day 210 upon revaccination on day 180. No changes in the geometric mean SNT_50_ were observed in the control arm of any of the groups.

**Figure 6:**
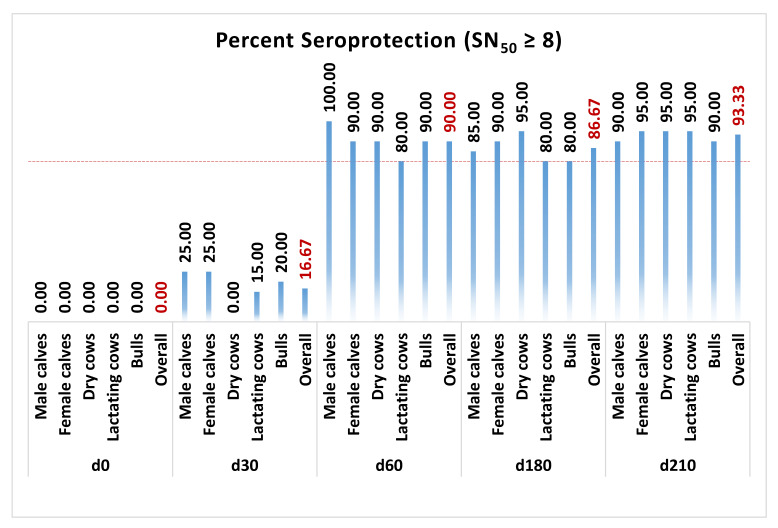
Group-wise seroprotection afforded by the vaccine. SNT_50_ ≥ 8 on day 60 was used as the cut-off for protection. A total of 90% of the subjects developed protective titers of SNT_50_ ≥ 8 after receiving both doses of initial vaccination and maintained protective titers until 180 days thereafter. Since the screening criterion was set at SNT_50_ < 2, seroconversion was defined as a four-fold rise in titers, and the seroprotection criterion was set at SNT_50_ ≥ 8; seroconversion was also equivalent to seroprotection.

Antibody titers of all five groups of animals, i.e., male calves, female calves, non-lactating cows, lactating cows, and breeding bulls, were determined on days 0, 30, 60, 180, and 210 of the trial (Figure 5). Following both doses of the primary vaccination at days 0 and 30, the geometric means of SNT_50_ in the treatment arms of all groups surpassed 8. Further, these titers persisted above 8 until revaccination, with the third dose on day 180. At Site A, three of the forty-five animals in the vaccine arm did not respond to the vaccine. Similarly, at Site B, two of the forty-five animals in the vaccine arm did not respond to the vaccine. One of forty-five animals at Site A and three of forty-five animals at Site B responded late only upon receiving revaccination with the third dose of the vaccine. In comparison, all control subjects at both trial sites showed SNT_50_ lower than 2 throughout the study until day 210. Overall, the percentage of subjects meeting or exceeding SNT_50_ ≥ 8 was 90.00%, 86.67%, and 93.33% on days 60, 180, and 210 of the trial, respectively (Figure 6).

## 4. Discussion

IBR is an economically important disease of animals. The greatest losses to Indian dairy farmers due to this disease are in the form of abortions, mostly occurring in the last trimester of pregnancy [22]. The majority of the cows suffering from vulvovaginitis show sharp declines in conception rate from 80% to 45–50% [23]. A non-fatal episode of bovine respiratory disease during calfhood has a lifelong impact on lung function and animal productivity, which may be the major source of disease-specific losses in feedlot cattle. A clinical episode of calfhood herpes pneumonia has been associated with an increase in age at first calving and reduced likelihood of completion of first lactation by heifers; even when the first lactation is completed, the milk yields are reduced [8]. The use of broad-spectrum antibiotics to prevent and treat the manifold secondary bacterial complications, including pneumonia, metritis, and mastitis [24,25], that commonly follow a disease episode makes BoHV-1 infection a major One Health problem globally.

Latency, after a clinical or subclinical infection, is an important epidemiological feature of most herpesvirus entities. Latency can be periodically reactivated upon infection with synergistic pathogens, stress, or other immunosuppressive conditions. The reactivated virus is trafficked to the site of initial infection and excreted, acting as a source of infection to other animals. Clinical signs may or may not accompany reactivation, and most seropositive animals bear latent infections [25].

First reported in the year 1976 by Mehrotra and co-workers [26], the disease has become enzootic in Indian cattle, and cumulative seroprevalence as high as 43% has been reported [14]. In a study involving 398 animals across four states of southern India, cattle having a history of abortions showed 100% seroprevalence for IBR [6]. Another study testing 1000 cattle sera samples from four different regions of India found IBR seropositivity in 65% retention of placenta cases, 76% of metritis cases, and 83% of the cases each of abortions and repeat breeding [7]. Studies from India’s adjoining countries have reported a seroprevalence of 47% in Tibet, 40% in overall China [27], 69% in the Lahore province of Pakistan [28], and more than 60% seroprevalence in Myanmar [29]. Besides cattle, seroprevalence has been reported from a wide variety of animals in India, including buffalo, yak, mithun, and elephant [30,31]. Goats and sheep can also be infected [32,33]. Buffaloes can get infected with both BoHV-1 and Bubaline Herpesvirus-1 (BuHV-1); BoHV-1 infection causes viremia and seropositivity without clinical disease in buffaloes. Antibodies to BoHV-1 are produced in buffaloes following an identical vaccination protocol used to immunize cattle [34]. Hence, vaccination of buffaloes against BoHV-1 should also be practiced in areas where they are co-reared with cattle to reduce contagion.

Key stakeholders have periodically voiced the need for a nationwide vaccination program against IBR in India with an inactivated marker vaccine [13,14]. However, the lack of an indigenous vaccine has proven limiting to the pursuit of such programs. Globally, both live attenuated and inactivated vaccines are available commercially for the control of the disease. Live attenuated vaccines protect against the severe form of the disease and reduce virus shedding post-infection [25] but have several problems. Naïve heifers receiving the live attenuated vaccine are more likely to suffer from abnormal estrous cycle and lower pregnancy rates as compared to heifers receiving the inactivated vaccine [35]. In another study on estrus cattle, virus shedding did not occur upon intramuscular inoculation of the live modified vaccine, but the conception rate was markedly decreased, contraindicating the intramuscular administration of live attenuated IBR vaccines during estrus in cattle [36].

Wild-type IBR virus causes severe histopathological changes in ovarian tissues upon intravenous inoculation on the day after breeding. Three different live vaccines, when given intravenously during estrus, also caused identical pathologic changes in the ovary [36]. Necrotic oophoritis was reported in heifers receiving a live vaccine [37]. Miller et al. could attribute embryonic deaths to live attenuated IBR vaccines even when administered up to two weeks after breeding [38]. Animals that receive a live vaccine can shed the virus in nasal secretions and in various bodily fluids such as semen, milk, and urine, thereby acting as a source of infection for susceptible animals [39,40]. Moreover, modified live vaccines have been reported to induce a post-vaccination reaction [40,41]. The use of a live vaccine is contraindicated in bulls producing semen due to the risk of spreading the disease if a latent virus is present [42]. A retrospective study attributed more than 95% of BoHV-1 fetal losses in the USA between the years 2009 and 2016 to live BoHV-1 vaccines [35].

Live IBR vaccines protect against BoHV-1-induced disease, but they do not protect against reactivation of latency and may themselves establish latency [43]. Historically, vaccinating individuals with live virus vaccines has been considered equivalent to natural infection in terms of setting up latency [44], and the high frequency (80 to 100%) of reactivation with live vaccines suggests that they are as good at inducing latent infections as the virulent virus [45]. In fact, several countries in Europe, including Austria, Denmark, Finland, Sweden, and Switzerland, had to prohibit vaccination with live vaccines to achieve BoHV-1 eradication [11].

Inactivated IBR vaccines, therefore, offer a non-abortifacient and non-latency alternative to live vaccine. Vaccination against BoHV-1 with killed vaccines can afford up to a 60% reduction in abortion risks [19]. However, inactivation reduces the effectiveness of the vaccine agent [46,47]. Many immunogenicity trials with inactivated IBR vaccines [48,49,50] have suggested that an optimum payload of inactivated viral antigen is necessary per dose of the vaccine for inducing adequate protection. Vaccines, especially of the inactivated type, containing antigen payloads lesser than 10^6.5^ TCID_50_ before inactivation are dangerous due to the risks of inadequate immunity resulting in silent virus shedders [32].

Besides inactivation, control of IBR also requires distinguishing naturally infected from vaccinated animals so that shedders can be culled or segregated. The use of traditional live or inactivated vaccines interferes with conventional serological diagnosis and makes epidemiological surveys difficult [51]. Hence, marker vaccines have been developed by removing the non-essential BoHV-1 proteins, most commonly glycoprotein-E (gE). Such vaccines allow the serological differentiation of antibodies between animals immunized with the marker vaccine and those with natural infection [25]. Vaccination with gE-deleted marker vaccines, followed by the removal of seropositive animals, allows countries with a high prevalence of BoHV-1 infections to run concurrent vaccination and eradication programs [5].

BoHV-1 has three serologically indistinguishable strains, viz. BoHV-1.1, BoHV-1.2a, and BoHV-1.2b. Initially, it was believed that BoHV-1.1 is associated with respiratory (IBR) and reproductive infections; BoHV-1.2a, frequently isolated from aborted fetuses, is associated with respiratory and genital infections; and BoHV-1.2b is associated with reproductive and neurological infections. However, it is now well accepted that the nature of disease caused depends more on the route of infection and management practices than on the subtype of the virus [52]. The virus causing IBR and the one causing IPV are one and the same agent [53,54], and the antigens against which host neutralizing antibodies are formed are relatively constant [52]. The ability to manifest a particular syndrome depends upon the mode of transmission rather than being the inherent property of the BoH-1 subtypes, and the same isolate produces a variety of different syndromes. BoHV-1 infects a wide variety of tissues without exhibiting specific tropism for either the respiratory tract or the genital tract, causing disease at the site of entry regardless of the virus strain. Nonetheless, differences do exist in the aggressiveness of the types; BoHV-1.1 is usually associated with the most severe forms of the disease and is excreted 10–100× more than BoHV-1.2b [33].

The present vaccine has been developed as an inactivated gE-deleted marker vaccine for IBR [15,16], which is best suited for control and eradication programs in a country like India and other Low- and Middle-Income Countries (LMICs), where prevalence is high and slaughter is either restricted or not advocated. Respective analytical methods based on the gE-deletion marker for serologically differentiating infected from vaccinated animals (DIVA) have also been developed but remain beyond the scope of this communication. The vaccine contains >10^8^ TCID_50_ of inactivated BoHV-1.1 per dose. Previously, the immunogenicity of the vaccine has been evaluated in guinea pigs and bovines, and the vaccine has been found to be immunogenic in both species, with protection in all animals until at least 180 days post-vaccination [16]. The present communication reports the results of the field trial of the said vaccine undertaken with the primary objective of assessing the safety of the vaccine. Secondary objectives of the trial included assessments of the immunogenicity of the vaccine and of the effect of the vaccine on the milk yield of lactating cows.

The trial was replicated at two sites across five physiological groups. The trial sites differed markedly in geoclimatic conditions, the genetic makeup of the animals, the characteristics of the breeds, and the farm practices. Animals at Site A consisted entirely of stall-fed Deoni breeds [55] of Indian cattle. Animals at Site B comprised almost equal numbers of Vechur breed [56] of Indian cattle and crossbreds (non-descript × HF/Jersey), reared under mixed farming, where the animals were stall-fed but also allowed to graze in open grasslands. The trial did not aim to establish breed differences in the reaction and response to the vaccine, but animals of different breeds and physiological statuses reared under contrasting geoclimatic and management conditions were included in the study so that the results of the trial were representative of, and could be generalized to, various types of cattle and rearing conditions. Internationally, IBR vaccines are recommended for use at six-month intervals following primary vaccination with two doses. Hence, the duration of the present trial was also kept at 210 days, including six months of assessment of the immune response following primary vaccination and another month following revaccination.

SNT was used for screening and assessing immune response to the vaccine. The primary immune response to BoHV-1 in cattle is characterized by the formation of IgM, IgG1, and IgA antibodies. Secondary immune responses primarily involve the formation of IgG2 antibodies [19,32]. SNT and competitive enzyme-linked immunosorbent assays (ELISAs) are commonly employed for the detection of BoHV-1 antibodies in bovine serum. ELISAs measure both neutralizing and non-neutralizing antibodies and may produce some false-positive results. This creates a range of uncertainty around the cut-off value, often requiring repeat testing [18,31]. SNT is specific for neutralizing antibodies and is recognized by the World Organization for Animal Health as the gold standard for the evaluation of immune responses as well as the standardization of other techniques [19,57].

Bovine trials are expensive and time-consuming, and finding seronegative animals adds to the difficulty of conducting open field trials in countries where infections are enzootic [58]. During screening, 10.67% (11 of 103) of animals tested positive at Site A, and 13% (10 of 77) of animals tested seropositive at Site B; cumulatively, 11.67% (21 of 180) of animals were seropositive. Trial Site A was situated in the state of Maharashtra, India, which has reported very high (64.95%) seroprevalence rates in the recent past [14]. It may be reasoned that being an organized dairy farm within a veterinary institution with good management and intensive farming practices, the herd has remained insulated from the disease to a greater extent. Trial Site B was situated in Wayanad district of the Indian state of Kerala. The seroprevalence rate recorded at Site B closely resembled the seroprevalence rates previously reported from the state, possibly because the animals grazed in open grasslands that were also visited by other domesticated, free-ranging, and feral cattle. Rajesh et al. studied the seroprevalence of IBR in the cattle population of Kerala, and 14.88% (107 of 719) of serum samples tested positive by Avidin Biotin Enzyme-Linked Immunosorbent Assay (AB-ELISA) [59]. Tresamol et al. conducted a cross-sectional study in the nearby Thrissur and Palakkad districts of Kerala during 2016–2017 to assess the seroprevalence of BoHV-1 in 517 cattle by AB-ELISA and found overall positivity of 10.63% [60].

In the current trial, the vaccine was tested in animals aged more than three months as per intended use. A syndesmochorial placentation in ruminants does not allow maternal gamma globulins to transfer to the fetus. Yet, many pre-colostral calves have anti-BoHV-1 antibodies, possibly due to transmission or leakage of maternal antibodies during pregnancy or oral intake of maternal blood at birth [61]. Vaccination of the neonatal calf is often recommended but can be limited in its efficacy in the face of high levels of maternally derived antibodies (MDAs). The vaccine antigen stimulates the immune system inadequately due to the masking effect of the MDAs, cytokines, and cells transferred from the dam to the calf in the colostrum [8]. Animals can usually be vaccinated after three months of age once MDA has depleted [32]. The average half-life of maternal antibodies (t_1/2_) for IBR has been shown to be 19 [62] to 21 days [63]. Animals do not seroconvert when vaccinated in the presence of persisting MDAs; nevertheless, such animals are primed for a secondary response, and they respond anamnestically upon subsequent vaccination at a time when MDA levels have decayed [63].

Inactivated IBR vaccines have typically been associated with frequent injection-site reactions and febrile reactions [32]. Trial Site A reported a pain-free, self-limiting, mild swelling at the site of vaccine injection in 2.9% (4 of 135) instances, which subsided uneventfully within 24 h without any medication. Site A did not report any other solicited or unsolicited adverse events or serious adverse events. Site B did not report any solicited or unsolicited adverse events or serious adverse events. No febrile reactions were observed, and the body temperatures of all subjects at both sites remained well below 102.5 °F at all the observation time-points; 102.5 °F is generally regarded as the upper threshold for normal body temperature of dairy cattle [64]. Following the second dose on day 30 of the trial at Site B, the mean rectal temperature of male calves was recorded to be negligibly lesser in the treatment arm (101.46 °F) than in the control arm (101.51 °F). Male dairy calves typically receive lesser care than female calves and may be more sensitive to even low levels of vaccination stress, which may have resulted in a slight lowering of body temperatures.

At Site A, the first dose was administered in dry, cool winters, the second dose in dry, hot summers, and the third dose in humid, warm rainy seasons. At Site B, the timing of the three doses corresponded to humid, warm winters; humid, cool summers; and humid, cool rainy seasons; respectively. Ambient temperatures are not known to directly impact the production of antibodies for IBR, although some influence on specific immune cells following vaccination is exerted [65]. Although the present study was not powered to do so, any season-specific differences in the antibody response to vaccination within or between the trial sites were not apparent.

A slight decrease in daily feed intake was observed at Site A for only 1–2 days in some groups following vaccination, and normal feeding resumed thereafter. Vaccination-induced stress is a well-known cause of transient decreases in the feed intake of vaccinated animals [66]. However, such stress and stress-associated changes are related to the process of vaccination rather than the vaccine itself. Site B did not record any significant differences in the mean intakes of the vaccinated treatment over the unvaccinated control arms in any of the groups following any of the three vaccinations. Further, neither of the trial sites recorded any changes in the behavior of feeding or rumination patterns of the animals.

Studies aiming to determine the effect of vaccines on milk yield must only consider short periods; measuring the average milk production over a long period fails to detect even significant short-term impacts [67]. Accordingly, milk yield was recorded for only five days prior and nine days following each of the three vaccination doses. At Site A, a slight numerical increase in milk yield was recorded following the third dose. Site B also recorded a non-significant increase in milk yield after the administration of the first and third doses. Although the contents of milk were not analyzed and the absolute volumes of milk were not corrected for contents, actual improvements in milk yield may be attributable to the immune stimulation caused by the vaccine [68]. More importantly, this observation affirmed that the vaccine did not induce considerable stress. The differences in the milk yield of the animals between the sites are attributable to the breed characteristics. Deoni cattle are poor yielders, and full-grown cows yielding about 1 L of milk per day are not uncommon [55]. Contrastingly, the local × HF/Jersey crossbreds at Site B were good yielders.

Antibody titers of all five groups of animals were determined on days 0, 30, 60, 180, and 210 of the trial. Since the screening criterion was set at SNT_50_ < 2, seroconversion was defined as a four-fold rise in titers, and the seroprotection criterion was set at SNT_50_ ≥ 8, seroconversion was equivalent to seroprotection. Following both doses of the primary vaccination at days 0 and 30, the geometric mean SNT_50_ in the treatment arms of all groups surpassed 8. Further, these titers persisted above 8 until revaccination with the third dose on day 180. Overall, the percentage of subjects meeting or exceeding SNT_50_ ≥ 8 was 90.00%, 86.67%, and 93.33% on days 60, 180, and 210 of the trial, respectively. Previously, Sarangi et al. [69] have also reported overall seroconversion of about 95% after five rounds of vaccination. Although statistically non-significant, the titers in calves and lactating cows were comparatively lesser than in non-lactating cows and bulls, which may be due to growth and production stress. As per standard requirements for potency of inactivated IBR vaccines laid down by the USDA, at least four of five (80%) susceptible male calves (SNT_50_ ≤ 2) should develop SNT_50_ ≥ 8 [20] at fourteen or more days after last vaccination. In our study, 20 of 20 (100%) susceptible male calves had SNT_50_ ≥ 8 after 30 days of receiving both doses of the primary vaccination, i.e., at day 60 of the study. Neutralizing antibodies are critical in preventing a BoHV-1 infection, and cell-mediated immunity is involved in recovery [70]. Although effective vaccines are expected to generate a neutralizing antibody titer of at least 8, immunization may protect despite the presence of any level of serum-neutralizing antibody due to sensitization of memory cells [19,62]. Specific antibodies are detectable from 10 days after exposure, and serum levels peak two to three weeks later [33]. The present study also observed peak titers at day 60, corresponding to 4 weeks after both doses of the primary vaccination. The titers decreased substantially by day 180, still above protective thresholds, and spiked markedly upon revaccination. Hence, the long-standing recommendation of repeating vaccination against IBR at six-month intervals [19,32] does appear valid.

## 5. Conclusions

Based on the findings of the trial, it could be concluded that the IIL-manufactured inactivated marker vaccine for IBR is safe for use in cattle of more than 03 months of age and does not cause elevation of body temperature, disturbances in feed intake, or any adverse effects on milk yield of lactating cows. Further, the vaccine affords protective immunity in cattle for up to a period of at least six months when administered with a primary vaccination regimen of two doses at monthly intervals. Overall, besides its favorable inactivated nature, DIVA capabilities, and superior antigen payload, the indigenously developed IBR vaccine also meets global standards of safety and immunogenicity.

## Figures and Tables

**Table 1:** Group-wise allocation of animals to vaccine and control arm.

Group	Vaccine Arm	Control Arm
Male calves	10	5
Female calves	10	5
Dry cows	10	5
Lactating cows	10	5
Breeding bulls	5	2

## Data Availability

The datasets analyzed during the current study are available from the corresponding author upon reasonable request.

## List of Abbreviations

AB-ELISA	Avidin Biotin Enzyme-Linked Immunosorbent Assay
ANOVA	Analysis of Variance
BoHV-1	Bovine Herpesvirus-1
DIVA	Differentiating infected from vaccinated animals
gE	Glycoprotein-E
HF	Holstein Friesian
IBR	Infectious Bovine Rhinotracheitis
IPB	Infectious Pustular Balanoposthitis
IPV	Infectious Pustular Vulvovaginitis
MDA	Maternally derived antibody
msl	Meters above sea level
SNT_50_	Median serum neutralization titer
TCID_50_	Median tissue culture infective dose
WOAH	World Organization for Animal Health
